# Structural and Comparative Analysis of the Complete Chloroplast Genome of *Pyrus hopeiensis*—“Wild Plants with a Tiny Population”—and Three Other *Pyrus* Species

**DOI:** 10.3390/ijms19103262

**Published:** 2018-10-20

**Authors:** Yongtan Li, Jun Zhang, Longfei Li, Lijuan Gao, Jintao Xu, Minsheng Yang

**Affiliations:** 1 Institute of Forest Biotechnology, Forestry College, Agricultural University of Hebei, Baoding 071000, China; liyongt37@126.com (Y.L.); zhangjunem@126.com (J.Z.); 2 Hebei Key Laboratory for Tree Genetic Resources and Forest Protection, Baoding 071000, China; 3 Changli Institute for Pomology, Hebei Academy of Agricultural and Forestry Science, Changli 066600, China; shv266@163.com (L.L.); gaolijuan0306@163.com (L.G.); swxujintao@aliyun.com (J.X.)

**Keywords:** *Pyrus hopeiensis*, cp genome, IR boundary, phylogeny

## Abstract

*Pyrus hopeiensis* is a valuable wild resource of *Pyrus* in the Rosaceae. Due to its limited distribution and population decline, it has been listed as one of the “wild plants with a tiny population” in China. To date, few studies have been conducted on *P. hopeiensis*. This paper offers a systematic review of *P. hopeiensis*, providing a basis for the conservation and restoration of *P. hopeiensis* resources. In this study, the chloroplast genomes of two different genotypes of *P. hopeiensis*, *P. ussuriensis* Maxin. cv. Jingbaili, *P. communis* L. cv. Early Red Comice, and *P. betulifolia* were sequenced, compared and analyzed. The two *P. hopeiensis* genotypes showed a typical tetrad chloroplast genome, including a pair of inverted repeats encoding the same but opposite direction sequences, a large single copy (LSC) region, and a small single copy (SSC) region. The length of the chloroplast genome of *P. hopeiensis* HB-1 was 159,935 bp, 46 bp longer than that of the chloroplast genome of *P. hopeiensis* HB-2. The lengths of the SSC and IR regions of the two *Pyrus* genotypes were identical, with the only difference present in the LSC region. The GC content was only 0.02% higher in *P. hopeiensis* HB-1. The structure and size of the chloroplast genome, the gene species, gene number, and GC content of *P. hopeiensis* were similar to those of the other three *Pyrus* species. The IR boundary of the two genotypes of *P. hopeiensis* showed a similar degree of expansion. To determine the evolutionary history of *P. hopeiensis* within the genus *Pyrus* and the Rosaceae, 57 common protein-coding genes from 36 Rosaceae species were analyzed. The phylogenetic tree showed a close relationship between the genera *Pyrus* and *Malus*, and the relationship between *P. hopeiensis* HB-1 and *P. hopeiensis* HB-2 was the closest.

## 1. Introduction

*Pyrus* belongs to the *Pyrus* ssp. of the Maloideae subfamily (Rosaceae), which mainly includes temperate fruit trees. There are more than 30 species in this genus, and 13 species are present in China [1,2]. The pear has been cultivated for over 3000 years in China. It is the third most commonly cultivated fruit tree after apple and citrus. China is the world’s largest pear producer, accounting for 71.2% of the world’s total pear area. Wild resources of *Pyrus* are extremely precious. They are mostly distributed in the valleys, hillsides, and forest margins, and are characterized by cold resistance, drought resistance, disease resistance, barren tolerance, saline–alkali tolerance, and strong adaptability. They provide material for screening quality rootstocks and for molecular breeding. The flowers, leaves, and fruit also have high ornamental value. The fruit of the pear is rich in fruit acids, vitamins, sugars, and many mineral elements essential for human life. It is sweet and refreshing and can be used to make dried and preserved pears, wine, and other products. Furthermore, it is regarded as having a high medicinal value and is used to reduce fevers, moisten the lungs, provide cough relief, and eliminate phlegm. Therefore, *Pyrus* is a valuable wild resource with a high exploitation value.

*P. hopeiensis* is a rare wild resource of the genus *Pyrus* in the subfamily Rosaceae [3,4], which has been listed as one of the “wild plants with a tiny population” in China. It can be found at the edges of hillside jungles at 100–800 m above sea level. At present, only a few genotypes have been found in Changli, Hebei Province. So far, there have been few studies about *P. hopeiensis*. Successful sequencing of the chloroplast genome of *P. hopeiensis* in our study provides a foundation for further study of its chloroplast molecular biology and can effectively promote genetic breeding and help clarify the molecular evolution of *P. hopeiensis*. It also provides some basis for the evolutionary analysis and classification of the genus *Pyrus*. What is more, this study gives a systematic review of *P. hopeiensis* that is useful for the conservation and restoration of wild *P. hopeiensis* resources.

Chloroplasts are the main site of photosynthesis, where fatty acids, starch, pigments, and other materials are synthesized [5]. They are independent of the plant nuclei and have a highly conserved genomic structure. Chloroplast DNA (cpDNA) has the beneficial characteristics of multiple copies, low molecular weight, and simple structure. Unlike the nuclear genome, which contains more repetitive sequences, and the mitochondrial genome, which is frequently rearranged, the chloroplast genome is rather conservative. The main mutation types are substitution and base insertion or deletion, and the mutation rate is low. Additionally, the chloroplast genome is of moderate size, making it easier to sequence than complex nuclear genomes. The chloroplast genome is maternally inherited in angiosperms with an independent evolutionary route [6]. Phylogenetic trees can be constructed using cpDNA data only, and the chloroplast genome shows good collinearity among plant groups. Sequencing data are relatively easy to analyze and the chloroplast genome structure sequence information can be used to study the species origin, evolution, and relationships between different species. In recent years, with the development of high-throughput sequencing technology, more chloroplast genomes of Rosaceae have been sequenced. In this study, the chloroplast genomes of two genotypes of *P. hopeiensis* and three other *Pyrus* (*P. ussuriensis* Maxin. cv. Jingbaili, *P. communis* L. cv. Early Red Comice, and *P. betulifolia*) were sequenced and compared with other Rosaceae plants. The genome structure and phylogeny of *Pyrus* were elucidated.

## 2. Results and Analysis

### 2.1. Basic Characteristics of Chloroplast Genome of P. hopeiensis

**Figure 1:**
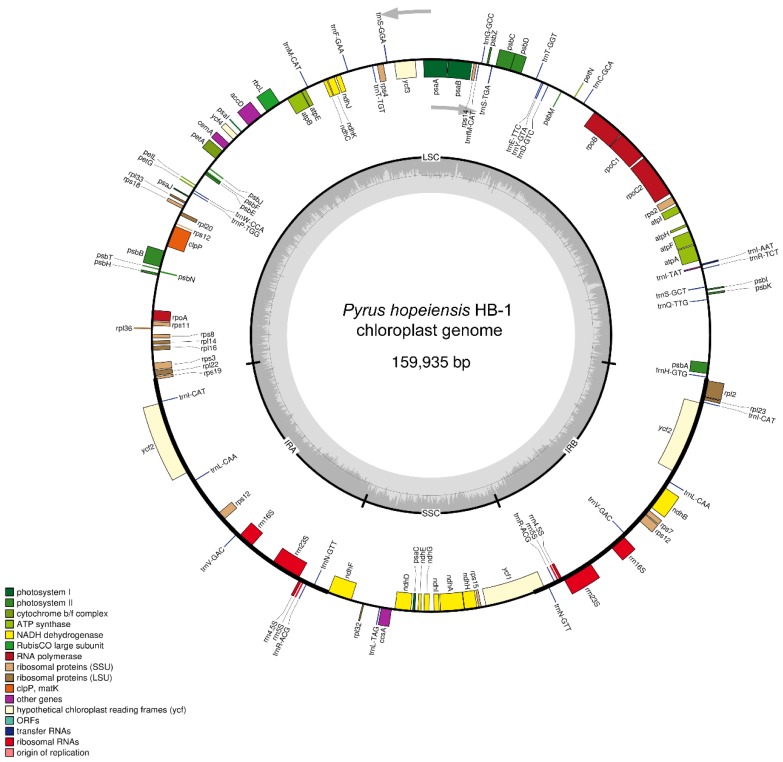
Gene maps of *Pyrus hopeiensis* HB-1chloroplast genomes.

The chloroplast genome of *P. hopeiensis* has a typical tetrad structure, including paired IRa and IRb sequences, encoding in opposite directions, and large and small single copy regions (Figure 1). The total chloroplast genome of *P. hopeiensis* HB-1 was 159,935 bp in length, 46 bp smaller than that of *P. hopeiensis* HB-2. The large single copy (LSC) region was 87,961 bp long, 46 bp smaller than that of *P. hopeiensis* HB-2. The length of the small single copy (SSC) region was 19,200 bp and the IR region was 26,387 bp, the same as those of *P. hopeiensis* HB-2. There was little difference in length between the two *P. hopeiensis* genotypes, and what difference existed was in the LSC region.

A total of 117 genes (Table 1) from the chloroplast genome of *P. hopeiensis* HB-1 were annotated, including 77 protein coding genes, 31 tRNAs, eight rRNAs, and two pseudogenes (*clpP* and *atpF*). *P. hopeiensis* HB-1 lacked only the *MATK* protein-coding gene that was associated with biosynthesis in *P. hopeiensis* HB-2. These 77 protein-coding genes can be divided into four categories. The first contains 28 self-replicating genes, including three subunits encoding the synthesis of chloroplast RNA polymerase. The second category contains 40 genes related to photosynthesis, including light systems I and II, a cytochrome b 6/f protein complex, and ATP synthase and other biosynthesis genes, including cytochrome-related genes (*P. hopeiensis* HB-1 contains three genes and *P. hopeiensis* HB-2 contains four genes). The fourth category contains five unknown genes, such as the *ycf* gene. The IR region of *P. hopeiensis* contains 32 genes, of which the *ndhB* gene is present only in the IRb region and is absent in the IRa region. In addition, *rps12* is a mitotic gene with its 5′ terminal located at the LSC region and 3′ end with a copy is located in each of the two IR regions. This phenomenon is common in higher plants.

The chloroplast genome of *P. hopeiensis* HB-1 contains 11 genes that harbor introns and one more *trnI-TAT* gene than *P. hopeiensis* HB-2. Of these 11 genes, two are tRNA genes (*trnI-TAT* and *trnI-AAT*) and nine are protein-coding genes (*rpoC1*, *rpl22*, *rpl22*, *ndhA, rpl2*, *rpl2*, *rps12*, *rps12*, *rps12*, *ycf3*), of which *ycf3* contains two introns. Excepting the intron length of the *trnI-AAT* gene, *P. hopeiensis* HB-2 is larger than *P. hopeiensis* HB-1. The exon and intron lengths of the two *P. hopeiensis* genotypes are identical. Among the coding genes, *ndhA* was the longest at 1125 bp, and rpl22 was the shortest at 63 bp.

### 2.2. Comparison of the Basic Characteristics of the Chloroplast Genome in Five Pyrus Species

The chloroplast genome of *Pyrus* is a typical ring structure 159,834–160,059 bp in length (Table 2). The chloroplast genome of *P. communis* L. cv. Early Red Comice is the shortest, and the longest is *P. ussuriensis* Maxin. cv. Jingbaili. The LSC length of *Pyrus* is 87,793–88,074 bp, the longest in *P. ussuriensis* Maxin. cv. Jingbaili and the shortest in *P. communis* L. cv. Early Red Comice. The SSC length is 19,201–19,261 bp, with the longest in *P. betulifolia* and the shortest in both genotypes of *P. hopeiensis*.

The IR regions were of similar length in four of the five *Pyrus* species, and only differed by 4 bp, except in *P. communis* L. cv. Early Red Comice. The GC content was similar, 36.57–36.59%. The number of protein-coding genes ranged from 74 to 78. The chloroplast genome of the five *Pyrus* species contained 15 genes, including introns (nine protein-coding genes and six tRNAs, see Table 3), and the *ycf3* gene contained two introns. The intron in *P. betulifolia* contained 13 genes, followed by 12 in *P. ussuriensis* Maxin. cv. Jingbaili, 11 in *P. hopeiensis* HB-1, 10 in *P. hopeiensis* HB-2, and 10 in *P. communis* L. cv. Early Red Comice. In the five *Pyrus* species, nine genes contained introns (*rpoC1*, *ycf3*, *rpl22*, *rpl2*, *ndhA*, *ndhB*, *rpl2*, *rps12*, and *rps12*), all of which were protein-coding genes. The intron in the *ndhA* gene was the longest, at 1125–1169 bp. Other than the short intron length of the *trnI-TAT* gene in *P. ussuriensis* Maxin. cv. Jingbaili, *rpl22* harbored the smallest intron of the other four *Pyrus* species, at 63–83 bp. The results showed that the size, structure, sequence, and GC content of the chloroplast genome of *P. hopeiensis* were similar to those of the other three *Pyrus* species, which was characteristic of the slow evolution of the genus *Pyrus* [7] in comparison to the chloroplast genomes of *P. hopeiensis*, *P. communis* L. cv. Early Red Comice, *P. ussuriensis* Maxin. cv. Jingbaili, and *P. betulifolia*.

### 2.3. Chloroplast Gene Gain–Loss Events

The chloroplast genome structure of most higher plants is stable, and the number, sequence, and composition of genes are conserved. However, the loss of chloroplast genome genes is common. For example, the chloroplast genome of the sweet orange has lost the *infA* gene [8]; the *ycf1*, *ycf2*, and *accD* genes have been lost in Gramineae [9], and the chloroplast genome of some legumes has been recombined several times, resulting in the deletion of a copy of the IR region [10]. The *rpl22* gene was detected in the chestnut nuclear genome [11], presumably having derived from the chloroplast genome. In addition, the *rpl32* gene was transferred to the nuclear genome in the poplar [12], and 17 similar chloroplast regions were found in the mitochondrial genome of papaya, suggesting that the chloroplast gene may have transferred to the mitochondria [13]. However, there have been few studies on how these genes are lost, transferred, and integrated into the nuclear and mitochondrial genomes.

In this study, we compared the gain–loss events of eight *Pyrus* species (five *Pyrus* species that were sequenced in this study and three other *Pyrus* species, *P. pyrifolia*, *P. spinose*, and *P. pashia*, which were downloaded from NCBI) (Table 4 and Table 5). The *psbL*, *psbl*, *trnI-GAU*, *trnA*, *trnL*, and *trnY-AUA* genes were most readily lost through evolution, followed by *trnA-UGC*, *trnG-UCC*, *trnI-AAU*, *trnI-GAU*, *trnK-UUU*, *trnN-AUU*, and *trnV-UAC*. *P. hopeiensis* HB-1 contained one less *MATK* gene than *P. hopeiensis* HB-2. Compared with the other three sequenced *Pyrus* species, *trnI-AAU* was only present in *P. hopeiensis* HB-1 and *P. hopeiensis* HB-2. The *atpB* gene was only lost in *P. ussuriensis* Maxin. cv. Jingbaili and *P. communis* L. cv. Early Red Comice, and *petB*, *petD*, *rps16* and *trnL-UAA* were present in the five *Pyrus* species sequenced here, but missing in *P. pyrifolia*, *P. spinosa*, and *P. pashia*.

### 2.4. Synonymous (KS) and Nonsynonymous (KA) Substitution Rate Analysis

Nucleotide mutations that do not cause amino acid changes are known as synonymous mutations, whereas nonsynonymous mutations do cause changes to the amino acid sequence. The Ka/Ks ratio (or dN/ds) of nonsynonymous substitution (Ka) and synonymous substitution (Ks) is the selection pressure of an encoded protein, which can be used to determine whether the gene encoded by the protein is under selection pressure. If Ka/Ks > 1, the protein is considered to be positively selected; if Ka/Ks = l, the protein is neutral; and if Ka/Ks < 1, the protein is considered to have undergone purifying selection. It is generally believed that synonymous mutations are not subject to natural selection, whereas nonsynonymous mutations are.

**Figure 2:**
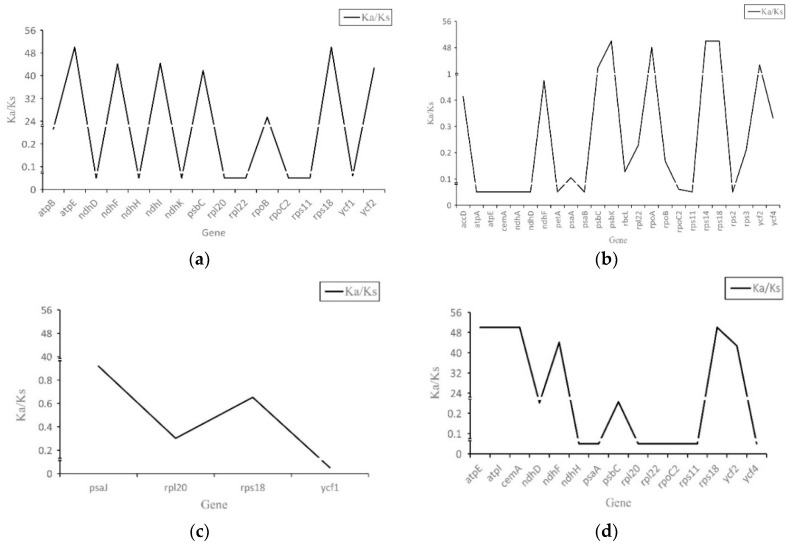
Ka/Ks value of five *Pyrus* species. (**a**)–(**d**) represent the Ka/Ks values of *Pyrus betulifolia*, *Pyrus communis* L. cv. Early Red Comice, *Pyrus ussuriensis* Maxin. cv. Jingbaili, and *Pyrus hopeiensis* HB-2, respectively, with respect to *Pyrus hopeiensis* HB-1.

Compared to *P. hopeiensis* HB-1, *psaJ*, *rpl20*, *rps18*, and *ycf1* in *P. hopeiensis* HB-2 were subject to negative selection, and no positive selection gene (Figure 2 and Table S1–S4) was found. In *P. betulifolia*, *atpE*, *ndhF*, *ndhI*, *rps18*, and *ycf2* were subject to positive selective pressure, whereas *ndhD*, *ndhH*, *ndhK*, *rpl20*, *rpl22*, *rpoC2*, *rps11*, and *ycf1* were subject to negative selection. The *psbC*, *psbK*, *rpoA*, *rps14*, *rps18*, and *ycf2* genes were subject to positive selective pressure in *P. communis* L. cv. Early Red Comice. Moreover, *accD*, *atpA*, *atpE*, *cemA*, *matK*, *ndhA*, *ndhD*, *ndhF*, *ndhH*, *petA*, *psaA*, *psaB*, *rbcL*, *rpl22*, *rpoB*, *rpoC2*, *rps11*, *rps2*, *rps3*, and *ycf4* were subject to negative selection. In *P. ussuriensis* Maxin. cv. Jingbaili, *atpE*, *atpI*, *cemA*, *ndhF*, *rps18*, and *ycf2* were subject to positive selective pressure, and *ndhD*, *ndhH*, *psaA*, *psbC*, *rpl20*, *rpl22*, *rpoC2*, *rps11*, and *ycf4* were subject to negative selection. Compared with *P. betulifolia*, *atpE* was subject to positive selective pressure in *P. betulifolia* and *P. ussuriensis* Maxin. cv. Jingbaili, whereas *atpE* was subject to negative selection in *P. communis* L. cv. Early Red Comice. This shows that the chloroplast genome of *Pyrus* has been affected by different environmental pressures during evolution, which may account for the different gene numbers among the five *Pyrus* species.

### 2.5. Indel Identification and Relationship of the Five Pyrus cp Genomes

The nucleotide bases in coding and non-coding regions have different evolutionary mutation rates. DNA variations located in coding regions can lead to large phenotypic and functional variations; moreover, these often have a slower mutation rate, making them suitable for phylogenetic studies of higher order elements (families, orders, and higher). Mutations in non-coding regions have little effect on phenotype and fewer functional restrictions, and as they take no part in the transcription/translation process, they have a relatively high nucleotide replacement rate and hence rapid evolution, making them suitable for the phylogenetic study of lower order elements (species, genus) [14].

**Figure 3:**
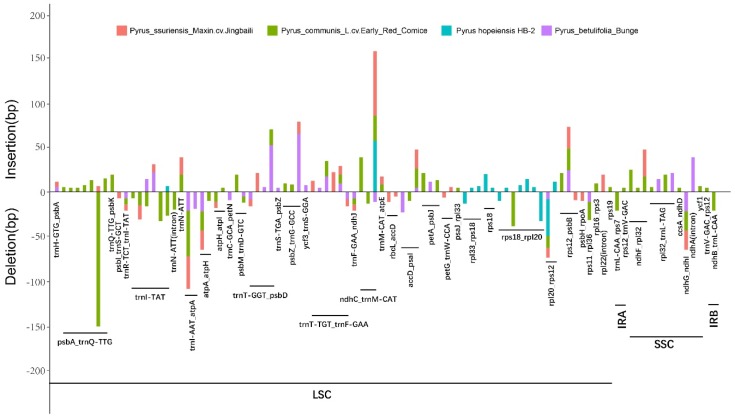
Indels (≥5 bp) identified based on multiple sequence alignment of five *Pyrus* cp genomes. Insertions are shown above and deletions below the horizontal axis. Indel distribution was positioned using *Pyrus hopeiensis* HB-1 as a reference.

The chloroplast genome data of five *Pyrus* species were compared with those of *P. hopeiensis* HB-1 by multiple sequence alignment using MAFFT. All differentially expressed sites were extracted using a script from the comparison results, and differences in sites of indels ≥ 5 bp were screened out. The location of different chloroplast genome sites was determined and ggplot in R was used to create graphic plots that were then optimized using AI. The results indicated 15 mutation sites in *P. hopeiensis* (Figure 3), which included 11 insertion and four deletion sites. All of these mutation sites were located in the LSC region; three were located in gene regions and 12 in intergenic regions. Among these, the longest was located in the *ndh*-*trnM-CAT* region, and as many as six mutations were located in the intergenic region *rpl18*–*rps20*. A total of 96 mutation sites were detected in the other four *Pyrus* species, 81 of which were located in the LSC region of the chloroplast genome and 11 in the SSC region, whereas only two mutation sites were found in the IRa and IRb regions in *P. communis* L. cv. Early Red Comice. There were more mutation sites in the SC region, and the IR region was more conserved. Indels were mainly located in the intergenic regions, and three indel loci were detected in the intron region (*rpl22*, *trnN-ATT*, *ndhA*). Because the protein-coding region is arranged by triplet codons, the tolerance of indels is poor. Therefore, only five indel loci were detected in the protein-coding region (*trnL-TAT*, *trnN-ATT*, *rps18*, *rps19* and *ycf1*), but no indel loci were detected in the rRNA region. A comparison of the occurrence of these indel loci among the four *Pyrus* species revealed 15 indel loci in the chloroplast genome of *P. hopeiensis*, 32 in *P. ussuriensis* Maxin. cv. Jingbaili, 57 in *P. communis* L. cv. Early Red Comice, and 31 in *P. betulifolia*. The insertion or deletion frequency in the chloroplast genome of *P. hopeiensis* HB-2 was less than that in *P. hopeiensis* HB-1. The *psbA*-*trnQ_TTG* and *rpl18*-*rps20* intergenic regions were the most variable regions with seven loci, followed by *trnT-TGT*_*trnF_GAA* (six) and the *trnI-TAT* gene-coding region (six). The largest indels were located in *psbA*_*trnQ-TTG* in the chloroplast genome of *P. communis* L. cv. Early Red Comice.

### 2.6. Codon Preference Analysis

Codons have an important role in the transmission of genetic information. Codon use is not equal in many species, and the phenomenon of a specific codon use frequency being higher than that of its synonymous codon is known as codon preference [15]. Codon preference is formed during the long-term evolution of organisms, with different species having different codon preferences. Codon use is affected by natural selection, mutagenesis, tRNA abundance, the composition of base groups, hydrophilicity of codons, gene length, and expression levels [16]. Analysis of the codon use preferences of a species improves our understanding of the transmission of genetic information and the development of evolutionary and phylogenetic models.

**Figure 4:**
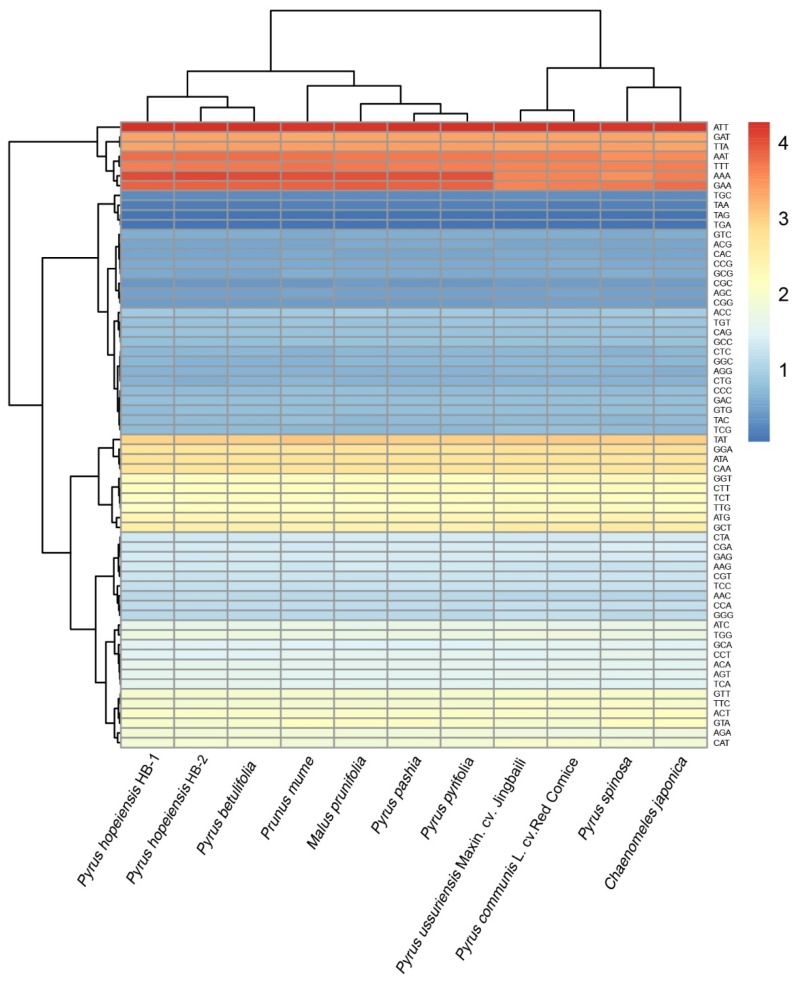
Codon distribution of all merged protein-coding genes. Red indicates a higher frequency and blue indicates a lower frequency.

The annotated files of plant genomes, including *P. pashia*, *P. pyrifolia*, *Malus prunifolia*, *Prunus mume*, and *Chaenomeles japonica*, were selected from the NCBI database, including the sequence files encoding CDS and proteins. According to the full-length CDS criterion, sequences with lengths <300 nt were deleted. The codon-use frequency of each genome was extracted from the annotated files of each genome and the corresponding frequency ratio was calculated. The final statistical results were clustered and mapped using the pheatmap package in R. The results showed obvious codon use preferences for both types of *P. hopeiensis*, among which ATT, AAA, GAA and AAT, and TTT were used most frequently (Figure 4). Statistical analysis of all the codons of *P. hopeiensis*, the three other *Pyrus* species, and the other Rosaceae showed a high A/T preference in the third chloroplast codon. This is common in the chloroplast genomes of higher plants [17,18,19,20,21].

### 2.7. Comparison of the Genome Structure in Rosaceae cp Genomes

The chloroplast genome structure of most higher plants is relatively stable and the number, sequence, and composition of their genes are conserved. However, because different plant groups have different evolutionary histories and genetic backgrounds, the chloroplast genome size, genome structure, and gene numbers vary. Insertion/deletion is the most frequent type of microstructural variation in the chloroplast genome, and it occurs frequently in some segments where the variation is high, such as *trnH*-*psbA* and *trnS-G*. In Rosaceae, an insertion/deletion of 277 bp in the intergenic region of the *trnS-G* gene was reported in peach plants [22], and an insertion/deletion of 198 bp in the intergenic region of *trnL-F* was identified in *P. mume* [23].

**Figure 5:**
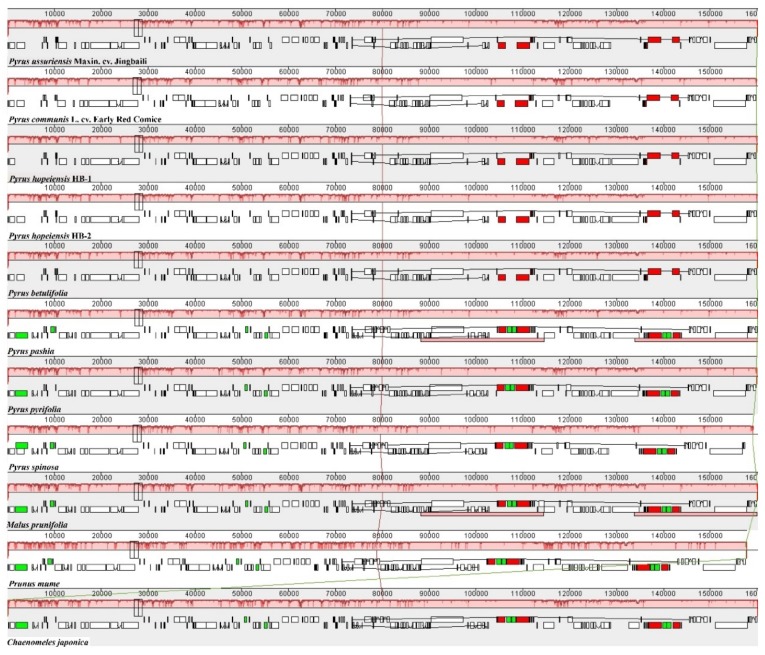
Co-linear analysis of various plant chloroplast genomes.

The collinear method was used to analyze and compare the chloroplast genomes of the two genotypes of *P. hopeiensis*, the other three sequenced *Pyrus*, and other related Rosaceae (*P. pashia*, *P. pyrifolia*, *P. spinosa*, *M. prunifolia*, *P. mume*, and *C. japonica*). The results showed optimal collinearity between *P. hopeiensis* HB-1 and *P. hopeiensis* HB-2, and only a few sites contained insertions and deletions (Figure 5). Compared with the other Rosaceae, the genome structure and gene sequences were highly conserved, with more linear relationships indicating high chloroplast genome homology among the different plants.

### 2.8. IR Contraction Analysis

The IR region is considered to be consistent and stable in the chloroplast genome. However, in the evolution of species, border region contraction and expansion are common. In this study, the IR boundaries of both genotypes of *P. hopeiensis* were compared. The IRa/LSC boundary extended into the *rps19* gene, and 120 bp of *rps19* extended into the IRa region. The IRa/SSC boundary extended into the *ndhF* gene, and 12 bp of *ndhF* extended into the IRa region. The IRb/SSC boundary extended into 1074 bp of *ycf1* and the IRb/LSC border extended into the *rpl2* gene, with the *trnH-GTG* gene located downstream.

**Figure 6:**
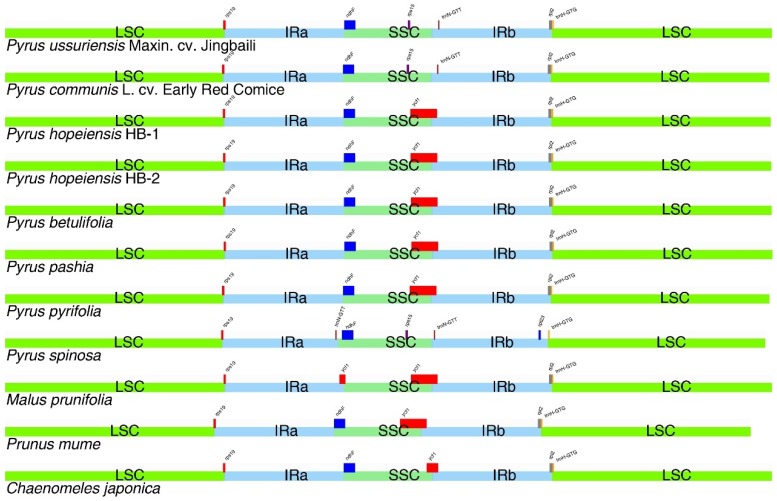
IR contraction analysis of Rosaceae.

The IR boundaries were compared among the Rosaceae, including the five *Pyrus* species sequenced, and *P. pashia*, *P. pyrifolia*, *P. spinosa*, *M. prunifolia*, *P. mume*, and *C. japonica* (Figure 6). The IRa/LSC boundary of these plants extended to the *rps19* gene. The IRa/SSC boundaries were located upstream of the *ndhF* gene, except in *M. prunifolia*, whose IRa/SSC junction lost *ndhF* but extended to *ycf1*. The *P. spinosa* IRb/LSC boundary had no *rpl2*. All IRb/SSC boundaries expect those of *P. ussuriensis* Maxin. cv. Jingbaili, *P. communis* L. cv. Early Red Comice, and *P. Spinosa* extended to *ycf1*. The IRb/SSC boundary lost *ycf1*. These findings were similar to those in the Actinidiaceae, Theaceae, and Primulaceae, but differed markedly from those in Ericaceae. For the IRb/LSC boundary, all but that of *P. spinosa* extended into the *rpl2* gene, and the IRb/LSC boundary of the *trnH-GTG* gene located downstream extended into the *rpl23* gene in *P. spinosa*.

### 2.9. Phylogenetic Analysis

**Figure 7:**
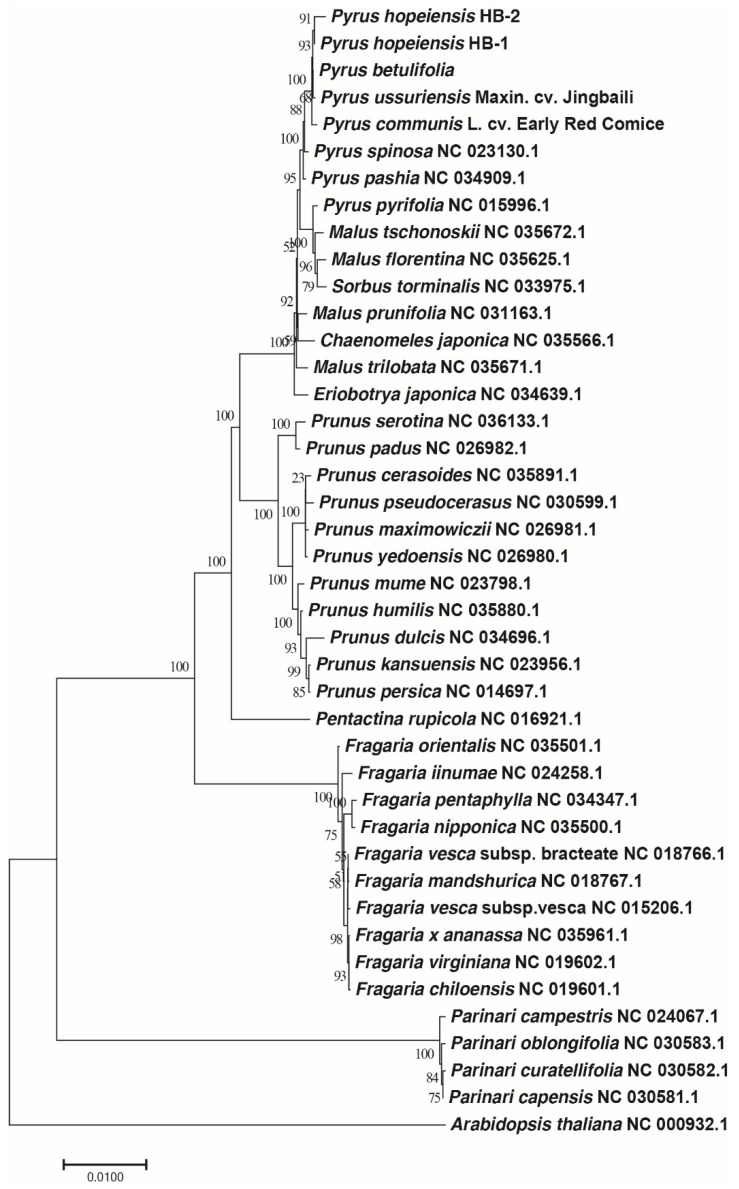
The ML phylogenetic tree of the Rosaceae clade based on same protein-coding genes. Numbers above or below the nodes are bootstrap support values.

To gain an insight into the position of *Pyrus* within the Rosaceae, a molecular phylogenetic tree was constructed using 57 protein-coding genes (*accD*, *atpA*, *atpE*, *atpH*, *atpI*, *csA*, *cemA*, *ndhA*, *ndhB*, *ndhC*, *ndhD*, *ndhE*, *ndhG*, *ndhH*, *ndhJ*, *ndhK*, *petA*, *petG*, *petL*, *petN*, *psaA*, *psaB*, *psaI*, *psbA*, *psbB*, *psbC*, *psbD*, *psbE*, *psbF*, *psbH*, *psbJ*, *psbM*, *psbN*, *psbT*, *rbcL*, *rpl14*, *rpl16*, *rpl2*, *rpl22*, *rpl23*, *rpl33*, *rpoA*, *rpoC1*, *rps11*, *rps12*, *rps14*, *rps15*, *rps18*, *rps19*, *rps2*, *rps3*, *rps4*, *rps7*, *rps8*, *ycf2*, *ycf3*, *ycf4*)from the chloroplast genomes of 36 Rosaceae, which were downloaded from GenBank, and using *Arabidopsis thaliana* as the outgroup. The resulting phylogenetic tree was consistent with the traditional plant morphological taxonomy (Figure 7), which can be divided into three sections: Maloideae, Prunoideae, and Rosoideae. The Maloideae include *Pyrus*, *Malus*, *Sorbus* L., and *Eriobotrya*. *Prunus* lies within the Prunoideae, and *Fragaria* is included in the Rosoideae. The phylogenetic relationship of the Prunoideae was closer than that of the Rosoideae to the Maloideae, and the relationship between *Malus* and *Pyrus* was the closest. Within *Pyrus*, the relationship between *P. hopeiensis* HB-1 and *P. hopeiensis* HB-2 was the closest, and the relationship between *P. betulifolia* and *P. ussuriensis* Maxin. cv. Jingbaili was closer than that between other *Pyrus* and *P. hopeiensis*. In addition, it can be seen from the evolutionary tree that Rosoideae is a subfamily that split off from the evolutionary tree.

## 3. Discussion

*Pyrus hopeiensis* is a valuable wild resource of *Pyrus*, which belongs to the family Rosaceae. Because of its limited distribution and population decline, *P. hopeiensis* is listed among “the wild plants with tiny population” in China. It belongs to one of 13 species of *Pyrus* present in China. In this study, the chloroplast genomes of the two genotypes *P. hopeiensis* HB-1 and *P. hopeiensis* HB-2 and those of three other major pear plants, *P. ussuriensis* Maxin. cv. Jingbaili, *P. communis* L. cv. Early Red Comice, and *P. betulifolia*, were analyzed using high-throughput sequencing for comparative analysis. The chloroplast genome of *Pyrus*, like those of most higher plants, is a typical tetrad consisting of two reverse repeat IR regions and small and large single copy fragments [24]. There was a 46 bp difference in the chloroplast genome size between the two *P. hopeiensis*, which was located in the LSC region. Compared with the other three *Pyrus* species, the total genome length was <225 bp, and the gene number, gene type, gene sequence, LSC, IR, and SSC lengths and GC content were similar. This strongly suggests that chloroplast genomes are highly conserved [25]. The encoded genes of the chloroplast genome are divided into three categories based on their functions. The first is related to chloroplast gene expression, such as tRNAs, rRNAs, and three subunits encoding chloroplast RNA polymerase synthesis. The second is related to photosynthesis, and the third consists of other biosynthesis genes and some genes of unknown function, such as mat and *ycf* [26]. The chloroplast genes of *Pyrus* are similar in composition.

The genomic sequence of the *P. hopeiensis* HB-1 chloroplast was used as a reference sequence to detect single-nucleotide polymorphisms (SNPs) and indels in the other four *Pyrus* species. The results showed a significantly higher variation in the non-coding region than in the coding region and more mutation sites in the intergenic region of the *psbA*-*trnQ_TTG*, *rpl18*-*rps20*, and *trnT-TGT*_*trnF_GAA* genes, which could be used for evolutionary analysis of *Pyrus*. The chloroplast genomes of *Pyrus* show obvious codon preference and similar codon use frequency. Furthermore, the third chloroplast codon has a higher A/T preference. This phenomenon is common in the chloroplast genomes of other higher plants [27]. Although the IR region is highly conserved, the expansion and contraction of the IR region is a common characteristic of the chloroplast genome. The degree of expansion of the IR/SC boundary is similar among the five *Pyrus* species, the two *P. hopeiensis* genotypes contain few genes with different extension positions, and any differences are very small, which is useful in the classification of *Pyrus*, as it can be used as a basis to identify the evolution of the chloroplast genome. The classification and identification of pear species in this study can be utilized for the preservation of pear germplasm resources. The initial identification of species and varieties of *Pyrus* was mainly based on morphological features (leaves, petioles, floral organs, sepals, hairs, fruits, and ventricles) and geographical distribution. For example, based on an investigation of morphological characteristics and natural distribution, Chinese taxonomists believe that *P. hopeiensis*, *P. phaeocarpa*, *P. sinkiangensis,* and *P. serrulata* were all formed by natural crosses [28]. Yu [29] divided *Pyrus* from China into 13 species based on their serrated leaf margins, and these included *P. hopeiensis*, *P. betulifolia*, *P. ussuriensis*, *P. phaeocarpa*, *P. bretschneideri*, *P. pyrifolia*, *P. pashia*, *P. armeniacaefolia*, *P. calleryana*, *P. pseudopashia*, *P. serrulata*, *P. sinkiangensis*, and *P. xerophila*. Anatomical studies in Wang Yingzhong [30] revealed that the anatomical structures of *P. betulifolia* and *P. ussuriensis*, and *P. bretschneideri*, *P. pyrifalia*, and *P. communis* were similar. The results showed that the relationship between *P. ussuriensis* and *P. betulifolia*, *P. bretschneideri,* and *P. pyrifalia* was close. However, it is easy to cross *Pyrus* species and there are no obvious differences in the biological and morphological characteristics among species and varieties, which greatly increases the difficulty of establishing its phylogenetic evolution and classification.

Pollen morphological identification, cytological markers, isozymes, and other methods have also been studied with a view to classifying *Pyrus*. The pollen morphology of *P. sinkiangensis* is similar to that of the Western pear, indicating a close relationship [31]. The pollen morphology of the Western pear is obviously different from that of the Oriental pear. The pollen morphology of *P. calleryana* has many primitive characteristics, and it is a primitive species of *Pyrus* in China. The pollen morphology of *P. bretschneideri*, also present in China, has the characteristics of both *P. pyrifalia* and *P. ussuriensis*, and may be a natural hybrid of *P. pyrifalia* and *P. ussuriensis*. Cytological markers enabled the analysis of the number, banding, karyotype, and meiosis behavior of the chromosomes. *P. phaeocarpa* has a similar karyotype to that of *P. betulifolia*, and those of *P. sinkiangensis*, *P. hopeiensis*, and *P. serrulata* were also similar [32,33]. In 1983, Lin Bonian and Shen Dexu [34] proved, through the use of the peroxidase isozyme, that *P. bretschneideri* and *P. pyrifolia* were closely related. However, these methods have few characteristic sites, poor polymorphism, and low accuracy, and provide a limited amount of information. To date, the relationships among *Pyrus* species, their origin, evolution of cultivation systems, and the origin of some suspicious species and hybrids remain unclear.

In recent years, molecular markers based on DNA, such as restriction fragment length polymorphisms (RFLPs) and simple sequence repeats (SSRs) have been used to investigate the genetic relationships, genetic diversity, and germplasm of *Pyrus*. However, there remain some deficiencies in the study of the interspecific relationships and origins of hybrids. Results based on random amplification of polymorphic DNA (RAPD) showed that the origin of *P. sinkiangensis* involved the crossing of many Eastern and Western pear species and that the genetic relationship between *P. bretschneideri* and *P. pyrifolia* is very close [35]. RAPD, inter sequence simple repeats (ISSR), and other DNA markers showed that *P. hopeiensis*, *P. betulifolia* and *P. phaeocarpa* were closely related to each other. In the same way, *P. phaeocarpa* is considered to be a hybrid of *P. betulifolia* and *P. ussuriensis*, whereas *P. hopeiensis* is a hybrid of *P. phaeocarpa* and *P. ussuriensis*. In a study using RAPD, *P. hopeiensis* and *P. phaeocarpa* shared some spectral bands with *P. betulifolia* and *P. ussuriensis* [36,37,38]. Zheng et al. identified a close relationship between *P. ussuriensis* and *P. hopeiensis* using internal transcribed spacer (ITS) sequences, which is consistent with the results in our study [39].

Because the chloroplast genome is the second-largest genome after the nuclear genome, it is maternally inherited in most angiosperms; thus it reflects the maternal evolutionary history, and this helps us to understand the maternal ancestors of suspected hybrids. The coding and non-coding regions of the chloroplast genome evolve at different rates, making them suitable for systematic research at different levels. The coding region is highly conserved and is only suitable for phylogenetic studies of families, orders, and higher taxonomic levels, whereas the non-coding regions are less constrained by function and the rapid evolutionary rate is suitable for plant phylogenetic studies at interspecific and subspecies levels. At present, the successful design of a set of universal primers for the chloroplast gene non-coding regions (such as *trnS*-*psbcc*, *trnL*-*trnF* and *accD*-*pasI*) [40] has made the study of chloroplast non-coding regions a hot topic in studies of the systematic relationship of *Pyrus*. Phylogenetic trees based on combinations of the sequences of *trnL*-*trnF* and *accD*-*psaI* in the chloroplast non-coding regions have further confirmed the theory of an independent evolution of the Oriental pear and the Western pear from the background of matrilineal evolution, and have shown the close relationship between *P. bretschneideri* and *P. pyrifolia* [41]. A study of the *trnL*-*trnF* region of cpDNA showed that *P. sinkiangensis* is closely related to the Western pear and the Oriental pear; the relationship between *P. betulifolia* and *P. ussuriensis* is close; *P. bretschneideri* is a hybrid of *P. ussuriensist*, *P. phaeocarpa*, and *P. pyrifolia*; and that the Western pear and Oriental pear are related to each other [42]. The sequences of these regions are highly conserved, with only limited sites available to provide information to unravel the phylogeny of *Pyrus*. However, no comprehensive and systematic cpDNA sequence analysis of *Pyrus* exists in China. To further our understanding of the inter-species relationships of *Pyrus* and to reveal the origin of hybrids and explore the evolutionary model of Eastern and Western pears, a wider range of representative species and varieties of Eastern and Western pears must be selected, and the nuclear gene fragments inherited by their parents should be combined, especially the low copy nuclear gene introns.

## 4. Materials and Methods

### 4.1. Plant Materials

In early May 2017, fresh leaves were collected from *P. hopeiensis* HB-1, *P. hopeiensis* HB-2, and three local *Pyrus* species, *P. ussuriensis* Maxin. cv. Jingbaili (which belongs to the *P. ussuriensis* family), *P. communis* L. cv. Early Red Comice (a high-quality variety of Western pear native to the United Kingdom) and *P. betulifolia* (most widely used in northern China as pear rootstocks) in Changli, Hebei Province and were stored before being transported to ORI-GENE Ltd., a science and technology company based in Beijing, China, for chloroplast genome sequencing.

### 4.2. DNA Sequencing, Genome Assembly, and Validation

The total DNA of fresh young leaves was extracted using a plant DNA extraction kit (Tiangen, Beijing, China). Agarose gel electrophoresis was used to detect DNA integrity, and purity and concentration were ascertained. The Illumina HiSeq platform was used to sequence the total DNA. After sequencing, the raw data was initially screened to remove low quality regions affecting the data quality and subsequent analysis needed to obtain the expected clean data. The SOAPdenovo2.01 [43] oligonucleotide analysis package was used to assemble the contig sequence. BLAT36 [44] was used to locate the assembled long sequence on the chloroplast reference genome of the relative species and to obtain the relative position of the contig sequence to enable splicing of the contig according to its relative position, and to correct assembly errors. A full-length frame map of the chloroplast genome was obtained. GapCloser software was used to fill gaps on the frame map sequence with high-quality short sequences. Any remaining gaps and suspected regions were supplemented and confirmed by generation sequencing, and the small single copy (SSC) and inverted repeat (IR) region junctions were verified. Finally, a complete ring chloroplast genome sequence was obtained.

### 4.3. Gene Annotation

CpGAVAS [45] was used to annotate the gene and the final annotation results were obtained by artificial correction. First, the results of Blastx, BLASTn, protein2genome, and est2genome [46] were integrated to predict the protein coding gene and the rRNA gene. Then, tRNA was identified using tRNAscan [47] and ARAGORN [48]. Finally, the reverse repeat region IR was identified using Vmatch [49]. Chloroplast genome mapping was performed using OrganellarGenomeDRAW [50] (http://ogdraw.mpimp-golm.mpg.de/index.shtml) based on the annotated results.

The protein and coding sequences (CDS) of each sample were extracted from the annotated files of each sample and the pairwise protein sequences aligned using MUSCLE software. The aligned protein sequences were converted to DNA sequences using PAL2NAL. KaKs_Calculator2.0 [51] software (https://sourceforge.net/projects/kakscalculator2) was then used to calculate Ka/Ks, which was used to analyze the selection pressure on different *Pyrus* species during the evolutionary process. Chloroplast genome sequences of 36 Rosaceae species were selected from NCBI and 57 common protein coding genes were used to explore the evolution of the *Pyrus* chloroplast genome, using *Arabidopsis thaliana* as the outgroup. The taxonomic status was confirmed. The annotated files of all of the genomes were downloaded from NCBI and the protein sequences of any genes shared among the chloroplast genomes of all of the species were extracted. Each gene was placed in a file in which each genome contained only one protein sequence. MUSCLE was used to make multiple sequence alignments for each file. The first and last sequences were aligned according to the genome source to obtain a growing alignment sequence: final.fa. MEGA7.0 software was then used to construct a neighbor-joining tree and the CGView Server was used to analyze the genetic variation among the chloroplast genomes of the five *Pyrus* species.

## 5. Conclusions

In this study, we reported the de novo sequencing results of *P. hopeiensis* chloroplast genomes. The length of the chloroplast genome of *P. hopeiensis* HB-1 is 159,935 bp, which is 46 bp longer than that of *P. hopeiensis* HB-2. The SSC and IR regions of the two Pyrus genotypes were the same length, with the only difference present in the LSC region. A total of 118 genes were identified in *P. hopeiensis* HB-1, and it only lacked the *MATK* protein-coding gene that was associated with biosynthesis in *P. hopeiensis* HB-2. The GC content of *P. hopeiensis* HB-1 was only 0.02% higher than that of *P. hopeiensis* HB-2. A total of 11 genes in the chloroplast genome of *P. hopeiensis* HB-1 contained introns, and an additional *trnI-TAT* gene not present in *P. hopeiensis* HB-2. *ycf3* is the only gene that contained two introns. The chloroplast genome structure and size, gene species, gene number, and GC content of *P. hopeiensis* were similar to those of the other three *Pyrus* species investigated. Almost all of the protein coding sequences and amino acid codons showed an obvious codon preference. Selection pressure analysis revealed that the chloroplast genomes of different pears were affected by different environmental pressures during the evolutionary process, which may account for the differences in gene numbers among the five *Pyrus* species. Phylogenetic analysis strongly supports the status of *Pyrus* in the Rosaceae. This study adds to our knowledge of the molecular evolution of *Pyrus*, and will be of use for the genetic breeding and chloroplast engineering of *Pyrus*.

## Figures and Tables

**Table 1:** Genes of the cp genome of *P. hopeiensis* HB-1.

Functions	Family Name	Code	List of Genes
Self-replication	Small subunit of ribosome	*rps*	*rps2*, *rps3*, *rps4*, *rps7*^a^, *rps8*, *rps11*, *rps12*^a^^b^^e^, *rps14*, *rps15*, *rps18*, *rps19*
	rRNA Genes	*rrn*	*rrn4.5S*^a^, *rrn5S*^a^, *rrn16S*^a^, *rrn23S*^a^
	Large subunit of ribosome	*rpl*	*rpl2*^a^^b^, *rpl14*, *rpl16*, *rpl20*, *rpl22*^b^, *rpl23*^a^, *rpl32*, *rpl33*, *rpl36*
	DNA dependent RNA polymerase	*rpo*	*rpoA*, *rpoB*, *rpoC1*^b^, *rpoC2*
	tRNA Genes	*trn*	*trnC-GCA*, *trnD-GTC*, *trnE-TTC*, *trnF-GAA*, *trnfM-CAT*, *trnG-GCC*, *trnH-GTG*, *trnI-TAT*^b^, *trnI-CAT*^a^, *trnI-AAT*^b^, *trnL-CAA*^a^, *trnL-TAG*, *trnM-CAT*, *trnN-GTT*^a^, *trnQ-TTG*, *trnP-TGG*, *trnR-TCT*, *trnR-ACG*^a^, *trnS-GCT*, *trnS-TGA*, *trnS-GGA*, *trnT-GGT*, *trnT-TGT*, *trnV-GAC*^a^, *trnW-CCA*, *trnY-GTA*
Genes for photosynthesis	Subunits of ATP synthase	*atp*	*atpA*, *atpB*, *atpE*, *atpF*^d^, *atpH*, *atpI*
	Subunits of protochlorophyllide reductase	*chl*	
	Subunits of NADH-dehydrogenase	*ndh*	*ndhA*^b^, *ndhB*^b^, *ndhC*, *ndhD*, *ndhE*, *ndhF*, *ndhG*, *ndhH*, *ndhI*, *ndhJ*, *ndhK*
	Subunits of cytochrome b/f complex	*pet*	*petA*, *petG*, *petL*, *petN*
	Subunits of photosystem Ⅰ	*psa*	*psaA, psaB*, *psaC*, *psaJ*, *psaI*
	Subunits of photosystem Ⅱ	*psb*	*psbA*, *psbB*, *psbC*, *psbD*, *psbE*, *psbF*, *psbH*, *psbI*, *psbJ*, *psbK*, *psbM*, *psbN*, *psbT*, *psbZ*
	Subunit of rubisco	*rbc*	*rbcL*
Other genes	Subunit of Acetyl-CoA-carboxylase	*acc*	*accD*
	Envelop membrane protein	*cem*	*cemA*
	c-type cytochrome synthesis gene	*ccs*	*ccsA*
	Protease	*clp*	*clpP* ^d^
	Translational initiation factor	*inf*	
	Maturase	*mat*	*matK*
	Elongation factor	*tuf*	
Genes of unknown function	Conserved open reading frames	*ycf*	*ycf1*, *ycf2*^a^, *ycf3*^c^, *ycf4*

^a^—Two gene copies in IRs; ^b^—Gene containing a single intron; ^c^—Gene containing two introns; ^d^—Pseudogene; ^e^—Gene divided into two independent transcription units.

**Table 2:** Comparison of the basic characteristics of chloroplast Genome in five *Pyrus* species.

	*Pyrus hopeiensis* HB-1	*Pyrus hopeiensis* HB-2	*Pyrus ussuriensis* Maxin. cv. Jingbaili	*Pyrus communis* L. cv. Early Red Comice	*Pyrus betulifolia*
Length (bp)	159,935	159,981	160,059	159,834	160,058
GC content (%)	36.59	36.57	36.57	36.58	36.57
AT content (%)	63.41	63.43	63.43	63.42	63.43
LSC length (bp)	87,962	88,008	88,075	87,794	88,025
SSC length (bp)	19,201	19,201	19,212	19,260	19,261
IR length (bp)	26,386	26,386	26,386	26,390	26,386
Gene number	118	119	117	114	120
Pseudogene number	2	2	2	2	2
Gene number in IR regions	32	32	31	31	32
Protein-coding gene number	77	78	75	74	77
Protein-coding gene (%)	64.25	65.55	64.10	64.91	64.17
rRNA gene number	8	8	8	8	8
rRNA (%)	6.78	6.72	6.84	7.02	6.67
tRNA gene number	31	31	32	30	33
tRNA (%)	26.27	26.05	27.35	26.32	26.50

**Table 3:** Statistics of gene introns in the chloroplast genome of five *Pyrus* species.

Gene	Strand	*Pyrus hopeiensis* HB-1	*Pyrus hopeiensis* HB-2	*Pyrus ussuriensis* Maxin. cv. Jingbaili	*Pyrus communis* L. cv. Early Red Comice	*Pyrus betulifolia*
*trnI-TAT*	−	√	×	√	×	√
*trnI-TAT*	+	×	×	√	×	√
*trnN-ATT*	+	×	×	√	√	×
*rpoC1*	−	√	√	√	√	√
*ycf3*	−	√	√	√	√	√
*rpl22*	−	√	√	√	√	√
*rpl2*	−	√	√	√	√	√
*ndhA*	−	√	√	√	√	√
*ndhB*	+	√	√	√	√	√
*rpl2*	+	√	√	√	√	√
*rps12*	−	√	√	√	√	√
*rps12*	+	√	√	√	√	√
*trnI-AAT*	+	√	√	×	×	×
*trnL-TAG*	−	×	×	×	×	√
*trnY-ATA*	+	×	×	×	×	√
Total	15	11	10	12	10	13

**Table 4:** Genes from the chloroplast genomes of *Pyrus*.

Specie	*atpB*	*matK*	*petB*	*petD*	*psaC*	*psbI*	*psbL*	*psbl*	*Rpl20*	*Rpl36*	*rps16*	*ycf1*	*trnI-GAU*	*trnA*
*Pyrus ussuriensis* Maxin. cv. Jingbaili	0	1	0	0	1	1	0	0	1	1	0	0	0	0
*Pyrus communis* L. cv. Early Red Comice	0	1	0	0	1	1	0	0	0	1	0	0	0	0
*Pyrus hopeiensis* HB-1	1	0	0	0	1	1	0	0	1	1	0	1	0	0
*Pyrus hopeiensis* HB-2	1	1	0	0	1	1	0	0	1	1	0	1	0	0
*Pyrus betulifolia*	1	1	0	0	1	1	0	0	1	1	0	1	0	0
*Pyrus pyrifolia*	1	1	1	1	0	0	0	1	1	0	1	1	1	1
*Pyrus spinosa*	1	1	1	1	1	1	0	0	1	1	1	1	0	0
*Pyrus pashia*	1	1	1	1	1	1	1	0	1	0	1	2	0	0
Total number of missing genes	2	1	5	5	1	1	7	7	1	2	5	2	7	7

**Table 5:** Genes from the chloroplast genomes of *Pyrus*.

Specie	*trnA-UGC*	*trnG-GCC*	*trnG-UCC*	*trnI-AAU*	*trnI-GAU*	*trnI-UAU*	*trnK-UUU*	*trnL*	*trnL-UAA*	*trnN-AUU*	*trnV-UAC*	*trnY-AUA*
*Pyrus ussuriensis* Maxin. cv. Jingbaili	0	1	0	0	0	2	0	0	0	1	0	0
*Pyrus communis* L. cv. Early Red Comice	0	1	0	0	0	0	0	0	0	1	0	0
*Pyrus hopeiensis* HB-1	0	1	0	1	0	1	0	0	0	0	0	0
*Pyrus hopeiensis* HB-2	0	1	0	1	0	1	0	0	0	0	0	0
*Pyrus betulifolia*	0	1	0	0	0	2	0	0	0	0	0	1
*Pyrus pyrifolia*	1	1	0	0	1	0	1	1	1	0	1	0
*Pyrus spinosa*	0	0	1	0	0	0	0	0	1	0	0	0
*Pyrus pashia*	2	1	1	0	2	0	1	0	1	0	1	0
Total number of missing genes	6	1	6	6	6	4	6	7	5	6	6	7

## Supplementary Materials

Supplementary materials can be found at http://www.mdpi.com/1422-0067/19/10/3262/s1.
