# Supplementary material for: Structural and Comparative Analysis of the Complete Chloroplast Genome of Pyrus hopeiensis—“Wild Plants with a Tiny Population”—and Three Other Pyrus Species
Source: Int J Mol Sci. 2018 Oct 20;19(10):3262. doi: 10.3390/ijms19103262 (PMC6214102; doi:10.3390/ijms19103262)
Supplement: Supplementary file 1 [file ijms-19-03262-s001.pdf]

Supplementary Tables

Table 1. The Ka, Ks, and Ka/Ks values of *Pyrus ussuriensis* Maxin. cv. Jingbaili with respect to *Pyrus hopeiensis* HB-1.

Gene	Ka	Ks	Ka/Ks
atpE	0.00330523	6.61E-05	50
atpI	0.00173843	3.48E-05	50
cemA	0.00173319	3.47E-05	50
ndhD	0.000813839	0.00322951	0.252001
ndhF	0.000507657	1.15E-05	44.1353
ndhH	4.03E-06	0.00403312	0.001
psaA	2.46E-06	0.00245945	0.001
psbC	0.000965312	0.00375455	0.257104
rpl20	1.33E-05	0.0133305	0.001
rpl22	2.01E-05	0.0200741	0.001
rpoC2	1.28E-06	0.00128285	0.001
rps11	1.36E-05	0.0135706	0.001
rps18	0.00965489	0.000193098	50
ycf2	0.000189159	4.43E-06	42.6809
ycf4	7.72E-06	0.00771876	0.001

Table 2. The Ka, Ks, and Ka/Ks values of *Pyrus hopeiensis* HB-2 with respect to *Pyrus hopeiensis* HB-1.

Gene	Ka	Ks	Ka/Ks
psaJ	0.975189	1.06165	0.918564
rpl20	0.139324	0.46208	0.301515
rps18	0.151566	0.232439	0.652066
ycf1	1.14E-06	0.00114463	0.001

Table 3. The Ka, Ks, and Ka/Ks values of *Pyrus betulifolia* with respect to *Pyrus hopeiensis* HB-1.

Gene	Ka	Ks	Ka/Ks
atpB	0.000850914	0.00320718	0.265315
atpE	0.00330523	6.61E-05	50
ndhD	6.02E-06	0.00602035	0.001
ndhF	0.000507657	1.15E-05	44.1353
ndhH	4.03E-06	0.00403312	0.001
ndhI	0.00246242	5.55E-05	44.3741
ndhK	9.65E-06	0.00964948	0.001
psbC	0.000826306	1.98E-05	41.832
rpl20	1.33E-05	0.0133305	0.001
rpl22	2.01E-05	0.0200741	0.001
rpoB	0.000415185	1.64E-05	25.3714
rpoC2	1.28E-06	0.00128285	0.001
rps11	1.36E-05	0.0135706	0.001
rps18	0.00453543	9.07E-05	50
ycf1	0.000209746	0.00348348	0.060212
ycf2	0.000189159	4.43E-06	42.6809

Table 4. The Ka, Ks, and Ka/Ks values of *Pyrus communis* L.cv.Early Red Comice with respect to *Pyrus hopeiensis* HB-1.

Gene	Ka	Ks	Ka/Ks
accD	0.00168934	0.00408468	0.413579
atpA	2.78E-06	0.00278319	0.001
atpE	1.04E-05	0.0104078	0.001
cemA	1.82E-05	0.0182325	0.001
ndhA	3.83E-06	0.00383102	0.001
ndhD	2.85E-06	0.0028529	0.001
ndhF	0.00268051	0.00282506	0.948835
petA	6.03E-06	0.00603266	0.001
psaA	0.000574263	0.00550034	0.104405
psaB	1.97E-06	0.00196951	0.001
psbC	0.000826306	1.98E-05	41.832
psbK	0.00632895	0.000126579	50
rbcL	0.00170222	0.0134876	0.126207
rpl22	0.00275821	0.0120906	0.228129
rpoA	0.00276728	5.76E-05	48.0785
rpoB	0.000384755	0.00230756	0.166737
rpoC2	0.000298614	0.00495215	0.0602999
rps11	1.36E-05	0.0135706	0.001
rps14	0.00413262	8.27E-05	50
rps18	0.00453543	9.07E-05	50
rps2	1.13E-05	0.0112847	0.001
rps3	0.00181912	0.00874131	0.208106
ycf2	0.000189159	4.43E-06	42.6809
ycf4	0.00255932	0.0077202	0.331509
